# Acetate containing sports beverages appear to alter buffering capacity during endurance exercise in trained male athletes

**DOI:** 10.1186/1550-2783-8-S1-P10

**Published:** 2011-11-07

**Authors:** Sylvia P Poulos, Tia M Rains, Mickey Rubin, Kathleen Kelley, Kevin C Maki

**Affiliations:** 1 Nutrition Research, The Coca-Cola Company, Atlanta, GA 30313, USA; 2 Biofortis-Provident Clinical Research, Glen Ellyn, IL 60137, USA

## Background

Acetate, a short chain fatty acid, is a metabolizableenergy source and may improve buffering capacity during exercise. Study objectives were to assess the effects of consuming beverages containing acetateon maximal anaerobic capacity, substrate metabolism, and total workoutput during timed endurance exercise.

## Methods

Trained male cyclists (n=11;24.3 ± 0.6 years; VO_2MAX:_54.9 ± 2.7ml/kg/min)consumed isocaloricsports beverages containing citric acid (placebo), triacetin (TRI), or acetic acid (AA)in a double-blind, randomized, controlled crossover study. Subjects consumed 710 mLbeverage anda standard breakfastbeginning each test day. Subjects performed two 30 second Wingate cycle tests separated by 4 minutes and consumed 7.5 ml/kg beveragewhile resting during a 60-min recovery period. Subjects then cycled for three 15-min consecutive segments at 65% VO_2MAX_ followed by a 15-min simulated “time trial.” Three ml/kg beverage was consumed during the endurance cycle test. Plasma glucose and lactate; serum free fatty acids, sodium, potassium, chloride, bicarbonate, osmolality; whole blood pH, urine osmolality and specific gravity were obtained at timesthroughout the day to assess markers of metabolism, and respiratory and cardiovascular variables were assessed during the time trial. Data were analyzed using repeated measures analysis of variance including subject and treatment as factors; Tukey’s test was used for pairwise comparisons. Data are presented as means ± SEM and p < 0.05 was considered significant.

## Results

There was no effect of beverage type on performance or blood markers of metabolism during the Wingate tests. During recovery, rating of perceived exertion was higher for TRI than AA (p = 0.03), systolic blood pressure was lower for TRI than AA (p = 0.03), and diastolic blood pressure was lower for TRI than AA(p = 0.04) and tended to be lower for AA (p = 0.07) than placebo.During the endurance test there were no significant effects of beverage type on blood markers of metabolism. Glucose decreased in all treatments after segment 1 and rebounded after segment 2. By the end of segment 4, glucose was higher than pre-endurance test levels in all treatments, and glucose tended to be higher with TRI compared toplacebo (p = 0.08). Lactate levels were generally lower during the endurance test in both acetate containing beverages versus placebo with a trend for TRI consumption to reduce lactate compared to placeboafter segment 3 (p = 0.06).There were no differences between treatments in respiratory and cardiovascular variables during the endurance test (p> 0.05). Minute ventilation was reduced with AAafter segment 3(p = 0.03), and triacetin (p = 0.08) versus control. Acetic acid consumption tended to reduce total work versus placebo (p = 0.06) during the time trial. There were no significant changes in urine specific gravity, urine osmolality levels, total urine volume, or net fluid loss throughout the day (p> 0.05).

## Conclusions

This study provides preliminary evidence to suggest that sports beverages containing acetate might have favorable effects on lactate and minute ventilation during submaximal endurance exercise in trained male athletes.

